# Proteomic Signatures of Multisystem Inflammatory Syndrome in Children (MIS-C) Associated with COVID-19: A Narrative Review

**DOI:** 10.3390/children11101174

**Published:** 2024-09-26

**Authors:** Maria-Myrto Dourdouna, Elizabeth-Barbara Tatsi, Vasiliki Syriopoulou, Athanasios Michos

**Affiliations:** Infectious Diseases and Chemotherapy Research Laboratory, First Department of Pediatrics, Medical School, National and Kapodistrian University of Athens, “Aghia Sophia” Children’s Hospital, 11527 Athens, Greece; myrtodour@med.uoa.gr (M.-M.D.); etatsi@med.uoa.gr (E.-B.T.); vsyriop@med.uoa.gr (V.S.)

**Keywords:** SARS-CoV-2, MIS-C, Kawasaki disease, omics, proteomics, biomarker

## Abstract

Background/Objectives: Multisystem Inflammatory Syndrome in Children (MIS-C) is a post-infectious complication of COVID-19. MIS-C has overlapping features with other pediatric inflammatory disorders including Kawasaki Disease (KD), Macrophage Activation Syndrome (MAS), Toxic Shock Syndrome and sepsis. The exact mechanisms responsible for the clinical overlap between MIS-C and these conditions remain unclear, and biomarkers that could distinguish MIS-C from its clinical mimics are lacking. This study aimed to provide an overview of how proteomic methods, like Mass Spectrometry (MS) and affinity-based proteomics, can offer a detailed understanding of pathophysiology and aid in the diagnosis and prognosis of MIS-C. Methods: A narrative review of relevant studies published up to July 2024 was conducted. Results: We identified 15 studies and summarized their key proteomic findings. These studies investigated the serum or plasma proteome of MIS-C patients using MS, Proximity Extension, or Aptamer-based assays. The studies associated the proteomic profile of MIS-C with laboratory and clinical parameters and/or compared it with that of other diseases including acute COVID-19, KD, MAS, pediatric rheumatic diseases, sepsis and myocarditis or pericarditis following COVID-19 mRNA immunization. Depending on the method and the control group, different proteins were increased or decreased in the MIS-C group. The limitations and challenges in MIS-C proteomic research are also discussed, and future research recommendations are provided. Conclusions: Although proteomics appear to be a promising approach for understanding the pathogenesis and uncovering candidate biomarkers in MIS-C, proteomic studies are still needed to recognize and validate biomarkers that could accurately discriminate MIS-C from its clinical mimics.

## 1. Introduction

Multisystem Inflammatory Syndrome in Children (MIS-C) is a rare, life-threatening post-infectious complication of COVID-19, occurring approximately 2–8 weeks after acute SARS-CoV-2 infection [1,2,3,4]. Affected children usually present with persistent fever, elevation of inflammatory markers, rash, gastrointestinal symptoms and/or cardiac complications, requiring hospitalization and, in severe cases, admission to the Pediatric Intensive Care Unit (PICU) [5,6]. MIS-C features overlap with the features of some other inflammatory and infectious pediatric conditions including Kawasaki Disease (KD), Macrophage Activation Syndrome (MAS), Toxic Shock Syndrome (TSS) and sepsis [1,6,7,8]. While it has been suggested that MIS-C is a result of immune dysregulation and hyperinflammation triggered by SARS-CoV-2 infection, the syndrome’s pathophysiology and the exact underlying mechanisms responsible for the clinical overlap between MIS-C and the above diseases remain incompletely understood [5,9,10,11]. In addition, the lack of biomarkers that could safely distinguish MIS-C from its clinical mimics complicates differential diagnosis, especially early in the disease course when establishing prompt and appropriate treatment is essential [1,9,10,11].

In this context, specific diagnostic and prognostic biomarkers are needed to differentiate MIS-C from other pediatric inflammatory conditions and guide clinical management. In recent decades, proteomic methods have evolved rapidly and have the potential to respond to this need for sensitive and specific MIS-C biomarkers [12]. Proteomics can provide extensive information about the structure and function of a large number of proteins, simultaneously [11,13]. Because of the recent advances in method development and bioinformatic analysis, the improvements in Mass Spectrometers and the increased availability of affinity-based proteomic techniques like Proximity Extension Assays (PEAs) and Aptamer-based methods, proteomic methods have been increasingly used in recent years to study various diseases, uncovering the underlying molecular mechanisms and enabling the identification of candidate biomarkers [14,15,16,17].

Despite these advances and given that MIS-C is a rare newly identified clinical entity, a relatively small number of studies that compared the proteomic profiles of patients diagnosed with MIS-C and patients with acute SARS-CoV-2 infection or conditions that mimic MIS-C, using Mass Spectrometry (MS), PEAs or Aptamer-based proteomic assays, have been published to date. Therefore, the implications of high-throughput proteomic technologies in the identification of MIS-C biomarkers are still unclear. In this review, we aimed to summarize the proteomic findings of these studies and provide an overview of how advanced proteomic technologies can offer an in-depth understanding of the pathophysiology and aid in the diagnosis and prognosis of MIS-C.

## 2. Methods

For this narrative review, a literature search was conducted at the PubMed electronic database to identify studies that compared the proteomic profiles of patients diagnosed with MIS-C and patients with acute SARS-CoV-2 infection or conditions that mimic MIS-C, using Mass Spectrometry (MS), PEAs or Aptamer-based proteomic assays. The keywords “MIS-C” Or “Multisystem Inflammatory Syndrome in Children” combined with the keywords “Proteomic” or “multi-omic” were used to identify relevant English language studies, published up to 31 July 2024. In addition, relevant studies from the references list of the eligible articles were also included.

## 3. Definition and Clinical Presentation of MIS-C

MIS-C was first described in April 2020, when a group of children presenting with hyperinflammatory shock and Kawasaki-like clinical features was reported in England [18]. A case definition was then implemented by the Centers of Disease Control and Prevention (CDC), which required, for the diagnosis of MIS-C, evidence of SARS-CoV-2 infection or exposure, clinical severe illness requiring hospitalization, the presence of fever for at least 24 h, the involvement of at least two systems (cardiovascular, gastrointestinal, renal, respiratory, hematologic, mucocutaneous, neurologic) and laboratory evidence of systemic inflammation [2,19]. While affected children can present with a diverse spectrum of signs and symptoms, fever, gastrointestinal symptoms (abdominal pain, vomiting, diarrhea) and cardiac manifestations are the most common findings [6,20]. Although, MIS-C is an uncommon complication of COVID-19, affecting <1% of SARS-CoV-2-infected children, it is a severe one, as up to 50% of children with MIS-C can present with shock, and approximately 50–80% have life-threatening illness requiring admission to the PICU [1,21].

Markedly, the clinical presentation of MIS-C overlaps with several pediatric inflammatory conditions including KD, MAS, sepsis and Staphylococcal or Streptococcal TSS [1,22]. The main clinical features of MIS-C and of the above conditions are summarized in Table 1. MIS-C and each one of these overlapping disorders can be severe and life threatening and therefore require early diagnosis and the administration of appropriate treatments. To improve specificity and reduce the risk of misclassification with other pediatric inflammatory disorders, the case definition of the syndrome from the CDC was updated in 2023 and differs with the 2020 definition in the following points: reduced number of organ/systems involved (the neurologic, respiratory and renal systems were removed), revised definitions of cardiac and hematologic involvement and establishment of a threshold for inflammation [C-reactive protein (CRP) ≥ 3 mg/dL] [2,19,23]. Additionally, in the revised MIS-C case definition, KD is considered to be an alternative diagnosis [2,19]. However, specific disease biomarkers for MIS-C are still lacking, making differential diagnosis complex [10].

## 4. Brief Overview of Relevant Proteomic Methods

Proteomics is the comprehensive study of the structure and function of proteins at a large-scale level [13]. The proteome is much more complex and dynamic than the genome, as alternative transcription, RNA splicing and post-translational modifications may lead to the generation of different proteins derived from the same genes affecting their function [33,34]. Therefore, in contrast with genomic analysis that can provide only a prediction of protein expression and function, proteomics can provide a more in-depth understanding of the underlying disease pathophysiology [35,36].

Due to technological advances in method development, in instrument speed, in the sensitivity, resolution and dynamic range of Mass Spectrometers and in bioinformatics, high-throughput proteomic methods have emerged in the last few years and have been used vastly for the discovery of biomarkers, with high sensitivity and specificity, for many diseases [14,15]. Currently, a variety of methods can be employed for the large-scale study of proteins, including MS, affinity-based proteomics and protein microarrays [35].

In this present review, we focus on MS-based proteomics and antibody- or aptamer-based affinity proteomic assays, as they are high-throughput methods commonly used in MIS-C biomarker research. Fifteen proteomic studies using MS or affinity-based proteomic assays to investigate the proteomic profiles of MIS-C patients were reviewed (Table 1) [5,9,10,11,37,38,39,40,41,42,43,44,45,46,47]. The characteristics of these studies and their key proteomic findings regarding patients with MIS-C compared to other pediatric inflammatory conditions are outlined in Table 2 and Table 3, respectively. The key proteomic findings regarding patients with MIS-C compared to healthy participants are presented in Supplementary Table S1.

### 4.1. Mass Spectrometry (MS)

MS is a high-throughput analytical detection technique that can determine the molecular weights and chemical structures of various biomolecules, including peptides and proteins [48,49]. MS-based proteomic approaches can be classified into “bottom-up” proteomics (also called “shotgun proteomics”) if the measurements were performed in peptides released from proteins after proteolysis and into “top-down proteomics” if intact proteins were measured [50]. Currently, “bottom-up” proteomics is considered to be the mainstay method for the discovery of *novel* disease biomarkers, enabling the analysis of thousands of different proteins in tissues or biological fluids [16,48,51,52]. In brief, proteins are digested into peptides using an endoproteinase with known specificity (such as trypsin), and the resulting peptide mixture is separated with Liquid Chromatography (LC), ionized and introduced to a Mass Spectrometer [34,48,52,53]. To identify the peptides and proteins from the MS data, software packages can be used to match the data to peptides form a relevant human protein database [16,52]. In addition to untargeted MS approaches that identify and quantify all detectable proteins in a sample, targeted approaches with MS-based assays can be applied for the quantification of preselected analytes [54,55]. Five MS-based studies, focusing on MIS-C biomarker research, are included in the current review (Table 2) [9,11,39,42,44].

### 4.2. Affinity-Based Proteomics

Over the last few years, scientific advancements have also enabled the development of high-throughput targeted proteomic assays using protein-specific affinity reagents [56,57]. Each one of the two most widely used techniques relays on a different affinity-based approach for the measurement of a large number of proteins [56]. Both techniques, while requiring a small amount of sample volume, are capable of identifying a large number of proteins in the sample [16].

The first affinity-based assay, Olink Proximity Extension Assay (PEA) (Olink Bioscience, Uppsala, Sweden) identifies proteins by using paired antibodies linked to complementary oligonucleotide sequences that can be then detected by Polymerase Chain Reaction (PCR) or Next Generation Sequencing (NGS) [16]. To date, several proteomic studies in MIS-C that used Olink’s PEA technology have been published [5,10,37,38,43,45,46,47]. The protein biomarker panels from the Olink platform that were used in these studies included the Target 96 Immune response, Target 96 Cardiovascular III, Target 96 Inflammation, Target 48 Cytokine panels and the Explore 3072 library (Table 2). The other method, aptamer-based SomaScan Technology (SomaLogic, Boulder, CO, USA), uses a library of highly specific, modified, short single-stranded oligonucleotides (DNA aptamers) as protein-binding reagents that are linked to fluorophores, which provide a quantification signal [16,56]. Two proteomic MIS-C studies that utilized this platform were included in this current review (Table 2) [40,41].

### 4.3. Biospecimens in MIS-C Proteomic Research

Regarding the type of biospecimen, all the proteomic studies included in this current review used either serum or plasma specimens. The serum or plasma proteomes consist of an attractive source for the discovery of diagnostic and prognostic disease biomarkers [58]. Not only is blood collection an inexpensive and minimally invasive medical procedure but also circulating proteins (from serum or plasma) are markers of systematic biological processes, and, therefore, they can be used to explore the underlying disease mechanisms [11,58]. Nevertheless, it should be noted that due to the complex composition of their proteome and the presence of high abundant proteins, the use of serum or plasma specimens in MS-based proteomic analysis is more challenging than the analysis of other types of specimens [48,59]. Markedly, the 20 most high abundant proteins (such as albumin and immunoglobulins) consist of 97% of the total plasma proteome mass, making the discovery of low- and very low-abundant proteins, like cytokines, difficult [59,60]. Various laboratory methods, like the depletion of the most abundant proteins and the removal of abundant proteins or peptides by chromatographic fragmentating, can be applied to overcome this issue [59]. However, in the depletion approach, low abundant proteins that specifically or non-specifically bind to the proteins targeted for depletion (e.g., albumin) can also be removed, and some of the methods may result in elongation of MS run time [15,16,59]. Therefore, the method of decreasing sample complexity should be carefully chosen [59].

## 5. MIS-C vs. Acute SARS-CoV-2 Infection

After SARS-CoV-2 viral infection, most children are either asymptomatic or experience mild and self-limiting COVID-19 symptoms [41,61]. To elucidate the differences between the underlying pathophysiological mechanisms of acute pediatric SARS-CoV-2 infection and of MIS-C, a number of studies investigated the protein alterations that occur during the course of MIS-C and compared them with those occurring during the acute phase of COVID-19 [38,41,42,43,44,45].

Utilizing multiplexed quantitative MS-based proteomics, Yonker et al. observed that in comparison to SARS-CoV-2-infected children, children with MIS-C had significantly higher levels of Zonulin and LPS-binding protein (LBP) [44]. As these proteins are markers of intestinal permeability, these findings may provide further insights in the pathogenesis of the syndrome [44]. Moreover, Druzak et al. studied the proteome of MIS-C patients with label-free LC-MS/MS and observed shared protein alterations compared to healthy controls and an upregulation of proteins involved in proinflammatory pathways, including changes in the complement and coagulation cascades, in the acute phase of SARS-CoV-2 infection and in MIS-C [42]. However, the shared alterations in protein expression levels were more significant in MIS-C, suggesting that MIS-C is a result of an over-exaggerated inflammatory response to SARS-CoV-2 [42]. Additionally, the KEGG’s (*Kyoto Encyclopedia of Genes and Genomes*) pathway analysis bioinformatic tool also revealed protein alterations that were unique to MIS-C (e.g., alterations in the biological processes of African trypanosomiasis and lipid metabolism) and to acute SARS-CoV-2 infection (e.g., alterations in various infectious processes including SARS-CoV-2 infection and metabolic processes related to nucleotides and proteins) (Table 3) [42].

Gruber et al. used a PEA (Inflammation panel of the Olink platform) to explore the plasma proteomes of children with MIS-C and with COVID-19 [43]. While the proteomic signature of MIS-C had similarities with that of SARS-CoV-2 infection, MIS-C patients had a unique cytokine profile that could separate them from patients with acute or convalescent SARS-CoV-2 infection [43]. Specifically, increased levels of various chemokines [C-X-C motif chemokine 1 (CXCL1), CXCL5, CXCL6, CXCL11] and cytokines [interleukin-17A (IL-17A), cluster of differentiation 40 (CD40), IL-6] were observed in the MIS-C group compared to the pediatric COVID-19 group (Table 3) [43]. In parallel, another PEA-based (Inflammation panel of the Olink platform) proteomic study also described differences in the proteomic profiles of children with MIS-C and acute SARS-CoV-2 infection, including an elevation of IL-10RB, CXCL9, IL-17A, vascular endothelial growth factor A (VEGFA) and a decrease in latency-associated Peptide (LAP), transforming growth factor beta-1 (TGF-beta-1) and Skp1/Cullin/F-box protein complex (SCF) in MIS-C (Table 3) [45].

Diorio et al., by employing an antibody-based protein biomarker platform (Olink Explore 1536/384), proposed that phospholipase A2 (PLA2G2A), a protein involved in host inflammatory responses, could be a candidate biomarker of MIS-C, as it could discriminate MIS-C patients from patients with acute SARS-CoV-2 infection [38]. In addition, the researchers found a correlation between the levels of PLA2G2A and some other proteins of the platform and the levels of soluble complement 5b9 (SC5b9) [38]. SC5b9 is a biomarker associated with Thrombotic Microangiopathy (TMA) [38]. TMAs are a group of rare, life-threatening diseases characterized by microangiopathic hemolytic anemia, thrombocytopenia and microthrombi that result in ischemic tissue injury [38,62]. The development of TMA has been reported as a potential complication of the hyperinflammation caused by SARS-CoV-2 infection in both adults and pediatric patients [63]. The activation of the complement cascade, specifically the alternative pathway, is a possible pathophysiological mechanism that leads to the development of TMA in the context of COVID-19 and MIS-C [63]. The above findings suggest that PLA2G2A could also associate MIS-C with clinical features of TMA [38]. Additionally, the researchers identified changes in the expression levels of several other proteins in MIS-C compared to patients with mild and severe SARS-CoV-2 infection (Table 3) [38].

Using SOMAscan technology, Sacco et al. observed that the activation of inflammatory process in children with MIS-C appeared to be more profound and qualitatively distinctive from those with acute SARS-CoV-2 infection [41]. More precisely, children with MIS-C had increased levels of several proteins including PLA2G2A, biomarkers of inflammation (e.g., IL-22, ferritin) and natriuretic peptide B (NPPB.1), among others, compared to children with SARS-CoV-2 infection (Table 3) [41]. The increased levels of NPPB.1 are consistent with the cardiac involvement observed during the course of MIS-C [41]. Notably, Gene Set Enrichment Analysis (GSEA) revealed hyperactivation of the matrisome-associated response, which comprises proteins associated with the extracellular matrix including the endothelium [41]. The activation of this pathway in combination with the increase in some other biomarkers associated with endothelial cell damage in this study may reflect the endothelial dysfunction that occurs in MIS-C and is also observed in other vasculitis syndromes including KD [41]. Changes in various other pathways were also observed in MIS-C compared to children with SARS-CoV-2 infection including, among others, the pathways of neurogenesis, neuron development and neuron differentiation (Table 3).

COVID-19-related Acute Respiratory Distress Syndrome (COVID-19-ARDS) is a more severe form of acute SARS-CoV-2 infection in children and is characterized by hypoxemic respiratory failure with bilateral lung infiltrates, multi-organ dysfunction and broad microthrombi formation [9]. A small discovery MS-based proteomic study aimed to determine the differences in plasma proteins between MIS-C and COVID-19-ARDS patients [9]. The researchers reported that while the proteomic profiles of MIS-C and COVID-19 ARDS patients did not separate distinctly, they generally differed from those of healthy controls [9]. In both conditions, alterations in the complement activation and coagulation pathways were observed, possibly indicating a shared underlying mechanism [9]. However, the MIS-C group was characterized by changes in FcGR and BCR activation pathways, while the COVID-19-ARDS group was characterized by alterations in the pathways of scavenging of heme and retinoid metabolism [9].

## 6. Association of Proteomic Signatures in MIS-C with Clinical Parameters

Regarding the clinical manifestations of MIS-C, Reiter et al. with the use of a PEA (Target 96 Inflammation and Cardiovascular III panels of the Olink platform), observed that the decreased serum levels of certain proteins [TNF-related subfamily member 9 (TNFRSF9) and apoptosis-inducing ligand (TRAIL)] were associated with cardiovascular manifestations and myocarditis in children with MIS-C [5]. Additionally, the researchers found that the levels of N-terminal prohormone of brain natriuretic peptide (NT-proBNP) and Chemokine (C-C motif) ligand 15 (CCL15) were higher in children with MIS-C related myocarditis [5].

To identify proteins associated with disease severity, a MS-based proteomic study found that 75 proteins including C-reactive protein (CRP), S100 calcium-binding protein A9 (S100A9), serum amyloid A(SAA) 1 (SAA1), SAA2, signal transducer and activator of transcription 3 (STAT3), low-affinity immunoglobulin gamma Fc region receptor III-A (FCGR3A), LBP, cluster of differentiation 163 (CD163), Alpha-1-acid glycoprotein 1 (ORM1), serpin family A (SERPINA) member 1 (SERPINA1), SERPINA3, Metallopeptidase Inhibitor 1(TIMP1), Talin-1 (TLN-1), Von Willebrand factor (VWF), various Immunoglobulins (Igs) and components of the C1 complex of the complement system were significantly upregulated, and 61 proteins were significantly reduced in severe MIS-C, compared with mild MIS-C [39]. The biological pathways in which the upregulated proteins in severe MIS-C were involved included the pathways of proteolysis, acute phase response, inflammation and coagulation pathways and the classical complement cascade [39]. By contrast, proteins involved in pathways, such as the negative regulation of peptidase activity, the pathway of extracellular matrix proteoglycans, the high-density lipid protein remodeling and in the complement and coagulation cascades, were downregulated in severe MIS-C [39].

Another proteomic study, carried out by Patel et al., identified significant correlations between protein expression in MIS-C and clinical variables including illness severity scores, interventions and hospital or PICU length of stay (LoS) by examining the plasma samples of MIS-C patients admitted to the PICU with a PEA (Olink Explore 3072 library) [10]. While several proteins were significantly associated with these parameters, significant correlations of four proteins were identified by both standard and COMBAT-Seq batch effect-adjusted feature selection [10]. These proteins included the following: leukotriene-A4 hydrolase (LTA4H) (neutrophil chemotaxis), pleiotrophin (PTN) (angiogenesis), pro-platelet basic protein (PPBP) and epidermal growth factor (EGF) (cell growth and differentiation). Specifically, in this study, the Pediatric Index of Mortality 2 (PIM2) score had a negative correlation with the expression of EGF [10]. Regarding interventions, Patel et al. reported that children with MIS-C, who were treated with inotropes or vasopressors, had higher levels of PTN than those that did not [10]. Hospital LoS had a positive correlation with LTA4H and PTN, while PICU LoS was inversely correlated with PPBP [10].

The administration of Intravenous Immunoglobulin (IVIG), which is also the mainstay of treatment in acute KD, and/or corticosteroids is the first-line treatment in children with MIS-C [64,65,66,67]. Second-line immunomodulatory therapy with biological agents [e.g., IL-1 receptor antagonist (anakinra)] may be also administered in refractory cases [64,68]. In addition, antithrombotic therapy is often used in MIS-C, and severe cases presenting with cardiorespiratory compromise or shock require additional supportive care [64,65]. Regarding response to treatment, a proteomic study carried out by Reiter et al. reported that the proteins whose expression was affected the most by treatment administration were mostly proteins involved in the pathways of neutrophil migration, chemotaxis and degranulation [5]. Furthermore, another proteomic study that assessed the cytokine changes upon treatment, also with the use of a PEA (Immune Response and Inflammation panel of the Olink platform), found that the levels of tumor necrosis factor beta (TNF-β), Integrin Subunit Alpha 11 (ITGA11) and CCL25 levels decreased, whereas the levels of Hexamethylene bisacetamide-inducible protein 1 (HEXIM1), Paraspeckle Component 1 (PSP1) and C-X-C motif chemokine ligand (CXCL)10 (CXCL10) increased in response to treatment [37]. As the researchers suggested, these proteins might be clinically useful markers to monitor the response of MIS-C patients to treatment [37].

## 7. MIS-C vs. Kawasaki Disease (KD)

KD is οne of the most common forms of pediatric vasculitis and is the leading cause of acquired heart disease during childhood in developed countries [28,69]. The diagnosis of classic KD is clinical and requires the presence of fever ≥5 days, and four out of five of the following clinical features: mucosal findings (erythema and cracking of lips, strawberry tongue and/or erythema of oral and pharyngeal mucosa), bilateral bulbar conjunctival injection without exudate, rash (maculopapular, diffuse erythroderma or erythema that is multiforme-like), erythema and edema of the hands and feet in the acute phase and/or periungual desquamation in the subacute phase and cervical lymphadenopathy (Table 1) [28]. Although an increase in several conventional inflammatory biomarkers, such as ESR, CRP and Procalcitonin, has been observed during the acute phase of KD, these biomarkers are largely non-specific. Several protein biomarkers like NT-proBNP, thrombospondin (TSP-1 and TSP-2), soluble suppression of tumorigenicity 2 (sST2) and clusterin have also been studied in KD, but most of them require validation from larger studies, and some of them may also be non-specific [70].

While MIS-C and KD have some discrete epidemiological, demographic, clinical and laboratory characteristics and are triggered by different infectious agents, the two entities have also many overlapping features [71]. Thus, the distinction of MIS-C from KD can be challenging for physicians.

To date, a small number of MS or affinity-based proteomic studies have explored the differences in the proteomes of these two clinical entities [5,37,39,46]. Porritt et al. performed a comparative proteomic analysis of patients with severe MIS-C and with KD using LC-MS [39]. The proteomic profiles of MIS-C and KD patients exhibited similarities, indicating common underlying pathogenic mechanisms between the two entities. However, several proteins that distinguished severe MIS-C from KD were also identified (Table 3) [39]. The expression of proteins involved in Ig-mediated immune activation [e.g., Fc Gamma Receptor IIIa FCGR3A], proteins associated with heart failure [e.g., Tenascin C (TNC), Quiescin sulfhydryl oxidase 1 (QSOX1)], was enhanced in severe MIS-C compared to KD [39]. These findings suggested that MIS-C could be mediated to a greater extent by immune complexes and that it is characterized by a more profound involvement of the heart muscle than KD [39].

Consiglio et al. examined the differences in the proteomic profile of children with MIS-C and KD by applying a PEA (Immune Response and Inflammation panels of the Olink panels) [37]. Although the researchers found similarities in the proteomic profiles of both groups of patients, they also recognized differences in the expression of some proteins, including IL-17A and Discoidin, CUB and LCCL domain-containing protein 2 (DCBLD2), which had lower levels in MIS-C compared to KD (Table 3) [37]. The functions of the proinflammatory cytokine IL-17A in the inflammatory process include the initiation of chemokine production, the activation of endothelial cells and the recruitment of neutrophils and monocytes to the site of inflammation [72]. It has been suggested that the IL-17A/IL-17 receptor A (IL-17RA) axis may play an important role in the severity of coronary arteritis in KD by mediating the aortic neutrophil chemoattraction [72]. Moreover, DCBLD2 is a protein secreted by endothelial and smooth muscle cells that is important for the regulation of angiogenesis, and its expression indicates the more profound arterial involvement in KD than MIS-C [37,73]. Overall, the elevation of biomarkers associated with arteritis and coronary artery involvement in KD comparison with MIS-C indicated that the endothelial involvement was more diffuse in MIS- C than in KD [37].

Additionally, Brodeur et al. used a 45-plex PEA (Target 48 Cytokine Panel of the Olink platform) and analyzed the plasma samples of patients with MIS-C and KD [46]. Besides some similarities in the cytokine profiles of KD and MIS-C, the researchers identified differences in a number of proinflammatory mediators associated with these syndromes (Table 3) [46]. Markedly, one or more members of the IL-17 cytokine family (IL-17A, IL-17C, IL-17F) were more elevated in KD compared to MIS-C [46]. The researchers suggested that IL-17 family cytokines, particularly IL-17A, could be hallmark biomarkers for KD, and might help discriminate KD from its clinical mimics including MIS-C. Notably, IL-17 cytokines were also associated with the development of Coronary Artery Aneurisms in KD patients [46]. As the researchers noted, the role of these cytokines as biomarkers for KD and potential risk factors for the development of Coronary Artery Aneurisms warrant further investigation [46]. In addition, the researchers also found that the cytokines IL-10, CCL4 and CCL8 were associated with MIS-C [46].

Another affinity-based proteomic study that also employed a PEA (Target 96 Inflammation and Target 96 Cardiovascular III panel of the Olink platform) revealed 84 unique protein markers that could discriminate treatment-naïve MIS-C patients from KD patients [5]. Regarding biomarkers discriminating MIS-C form KD, the authors found little agreement with the two aforementioned PEA-based markers studies, among the 19 biomarkers commonly analyzed in all three studies [5,37,46]. Notably, in this study, IL-17A and IL-17C were not elevated in KD compared to MIS-C [5]. As the authors suggested, these contrasting findings may be due to the rather small number of treatment-naïve KD participants included in their study, as IVIG treatment may effect IL-17A-expressing T-cells [5]. Nevertheless, they reported that the overexpression of IL-17A separated both MIS-C and KD from secondary Hemophagocytic Lymphohistiocytosis (sHLH)/Macrophage Activation Syndrome (MAS) [5].

## 8. MIS-C vs. Secondary Hemophagocytic Lymphohistiocytosis (sHLH)/Macrophage Activation Syndrome (MAS) 

MAS is another condition that may present with similar clinical and laboratory features to MIS-C [74]. MAS is a potentially life-threatening complication of various inflammatory conditions, caused by the pathological hyperactivation of T-lymphocytes and macrophages that have an hemophagocytic activity [75,76]. MAS is considered to be a secondary form of HLH (sHLH) associated with autoimmune disorders, particularly systematic Juvenile Idiopathic Arthritis (s-JIA) [75]. Fever and hepatosplenomegaly are the main clinical features of the syndrome followed by coagulopathy and circulatory, respiratory and multiorgan failure [75]. Findings from the laboratory evaluation of patients with MAS include hyperferritinemia, thrombocytopenia, hypertriglyceridemia, decrease in the Erythrocyte Sedimentation Rate (ESR), pancytopenia, increased liver enzymes and hypofibrinogenemia [77]. Some important cytokine biomarkers that have been studied for the diagnosis, activity and prognosis of s-JIA and MAS include IL-1, IL-1β, IL-10, Il-18, interferon-gamma (IFN-γ) and ΤΝF-a. [78] MAS can also be a complication of MIS-C [75]. MAS should be suspected in MIS-C cases refractory to treatment, and appropriate treatment should be initiated immediately, as it could be fatal if the diagnosis is delayed [75].

Besides patients with KD and MIS-C, Brodeur et al. contacted a high-fidelity proteomic analysis using a PEA (Target 48 Cytokine Panel of the Olink platform) to characterize the proteomic profile of patients with other pediatric rheumatic diseases, including MAS [46]. This study identified an elevation of IL-10, CCL4 and CCL8 in MIS-C compared not only to KD but also to MAS [46]. In both patients with MIS-C and patients with MAS, an elevation of IFN-γ and CXCL9 was detected compared to the rest of the participants, but the elevation of IL-33 was a distinguishing feature of patients with MAS [46]. In addition, higher levels of IL-18 in patients were detected in patients with and MAS (Table 3) [46]. Moreover, Reiter et al. by utilizing a PEA, recognized 58 differentially expressed proteins that could separate MIS-C patients from patients with sHLH/MAS (Table 3) [5]. An overexpression of IL-18 in the sHLH/MAS group compared to the MIS-C group was also reported in this study [5]. Still, IL-18 was also elevated in the MIS-C group, reflecting the hyperinflammatory state of the syndrome [5]. Markedly, the researchers observed more similarities in the proteomic signatures of MIS-C and sHLH/MAS compared to MIS-C and KD [5].

With the hypothesis that thrombocytopenic patients with MIS-C may have a more distinct HLH phenotype, Tulling et al. compared the proteomic profiles of thrombocytopenic and non-thrombocytopenic children diagnosed with MIS-C by applying a custom 60 protein panel Luminex assay and validating the results with a PEA (3072-plex panel of Olink platform) in an external cohort of MIS-C patients [47]. The researchers found that the proteomic profile of MIS-C patients exhibiting this HLH-like phenotype was characterized by more profound hyperinflammation, mainly driven by hallmark HLH-related serum biomarkers (soluble IL-2RA, ΤΝF-α, IFN-γ) [47]. Specifically, the levels of soluble IL-2RA (T-cell activation marker) were increased in thrombocytopenic children compared to non-thrombocytopenic children [47]. An inverse correlation between markers of T-cell activation, TNF-α and IFN-γ signaling and the levels of thrombocytes was observed, suggesting that thrombocytopenic children are characterized by a more exaggerated cytokine release [47]. Moreover, genomic analysis of MIS-C patients (with or without thrombocytopenia) was also contacted with Whole Exome Sequencing (WES) to identify gene mutations related to primary HLH [47]. The researchers did not recognize MIS-C patients with pathogenic variants of primary HLH-related genes in either of the MIS-C patient groups (with or without thrombocytopenia) [47]. Diorio et al. compared the proteomic profiles with a PEA of MIS-C patients that also fulfilled the Ravelli MAS criteria and those that did not and found that the following MAS-associated proteins were significantly elevated in MIS-C patients who also met the MAS criteria: CD163 (macrophage activation), CXCL9 (IFN-γ signaling), IFN-γ, IL-2RA (T-cell activation) and v-set and immunoglobulin-domain containing protein (VSIG4) [27,38]. Collectively the findings of these two studies demonstrated that the proteomic signatures of a distinct subset of MIS-C patients overlap with those of MAS/HLH [38,47].

## 9. MIS-C vs. Pediatric Infection

### 9.1. MIS-C vs. Bacterial and Viral Infection

Nygaard et al. used state-of-the-art unbiased MS-based proteomics and identified 105 proteins that had significantly different levels in the plasma of children diagnosed with MIS-C compared to children with other pediatric febrile conditions including bacterial infection, viral infection, severe sepsis and KD [11]. Most of the identified proteins were proteins involved in the biological processes of immune response (innate or adaptive), coagulation, lipid metabolism, cell death and growth (Table 3) [11].

As the authors suggest, the alterations in proteins involved in the coagulation cascade may explain the higher incidence of hypercoagulability and the increased risk of thromboembolic events in patients with MIS-C [11,79]. Furthermore, possible explanations for the significant changes that were observed in the lipidemic profiles of MIS-C patients are the function of lipid mediators as proinflammatory mediators and their role in vasodilation and vascular leakage, a frequent and severe clinical feature of MIS-C [11]. Additionally, the profound changes in proteins involved in cell death, growth and/or remodeling reflect the dysregulation of cellular and immune processes and the multiorgan involvement observed in MIS-C [11].

Moreover, by further analyzing patients’ proteomic data with artificial intelligence (AI), the researchers developed a highly accurate four-protein diagnostic signature, including Lymphocyte Cytosolic Protein (LCP1), Fc Gamma Receptor IIIa (FCGR3A also known as CD16a), Alpha-1-antichymotrypsin (SERPINA3) and Butyrylcholinesterase (BCHE), that could discriminate children with MIS-C from children with bacterial and viral infections [Area Under the Curve (AUC): 100%] [11]. Specifically, the levels of LCP1, CD16a, and SERPINA3, which are involved in immune dysregulation, were increased, whereas the levels of BCHE, which is involved in the hydrolysis of chlorine esters, were reduced in the MIS-C group (Table 3) [11]. It should be noted that the role of BCHE in the pathogenesis of the syndrome remains unknown [11]. Collectively, the above findings suggest that MIS-C is an intricate immunometabolic condition characterized by global hypercoagulability [11].

### 9.2. MIS-C vs. Pediatric Sepsis

Another severe pediatric condition that often mimics the clinical presentation of MIS-C, is sepsis [1]. Sepsis is a result of a dysregulated inflammatory host response to severe infection [1,30]. To this day, sepsis remains a significant cause of morbidity and mortality in children worldwide, as it accounts for >8% of PICU admissions and >4.5 million childhood deaths per year [1,30]. As with MIS-C, children with sepsis may also present with persistent fever, hypotension, shock or multiorgan dysfunction [1]. Similar to MIS-C, children with sepsis also have increased levels of inflammatory biomarkers [1]. Inflammatory biomarkers that have been studied and used in pediatric sepsis are CRP, procalcitonin, IL-6 and IL-8 [80]. Promising markers of pediatric sepsis also include the human neutrophil gelatinase (NGAL) and proadrenomedullin (proADM) [80].

However, the treatments of these two clinical entities differ in many aspects, especially considering that, in sepsis, the early initialization of antimicrobial therapy is associated with improved survival [1,30]. Therefore, given that prompt diagnosis and administration of specific treatments are required in both MIS-C and pediatric sepsis, distinguishing the syndromes early in the disease course is of utmost importance [1,10].

Patel et al. applied a PEA (Olink Explore 3072 library) to analyze the plasma proteome of children with MIS-C and children with SARS-CoV-2-negative sepsis and demonstrated that the plasma proteome of children with MIS-C was distinct from that of septic children [10]. Specifically, of the 2870 unique blood proteins, 58 differentially expressed proteins were identified with feature selection [10]. The identified proteins are involved in various biological processes including inflammation, metabolism, angiogenesis, cell growth and survival, and organ/cell-specific functions [10]. By using Natural Language Possessing for organ system and cell type expression pattern analysis, the researchers found that the 58-protein set was expressed in all organ systems with the highest levels of expression in the gastrointestinal system, and the most involved cell types were leukocytes, lymphocytes, macrophages and platelets [10]. Fifteen proteins, included in the 58-protein set, were also identified with COMBAT-Seq batch effect-adjusted feature selection as important candidate biomarkers for distinguishing sepsis from MIS-C (Table 3) [10]. This 15-protein set included proteins expressed in different organ systems [10]. For example, Angiopoietin 1 (ANGPT1) is a primary regulator of angiogenesis, PPBP is released from activated platelets and is associated with the risk of thrombosis and CREB/ATF BZIP Transcription Factor (CREBZF) is a protein that is expressed in the liver, kidney and pancreas and was decreased in MIS-C patients [10]. Brain-derived neurotrophic factor (BDNF) an important synaptic protein associated with neuronal survival, plasticity and signaling, was included in this set of 15 proteins and was found to be increased in MIS-C patients, indicating that the nervous system was affected in these patients [10].

## 10. MIS-C vs. Cardiac Adverse Events Following COVID-19 Immunization

Very rarely, cardiac adverse events, including myocarditis and pericarditis, have been reported after the administration of mRNA COVID-19 vaccines [81,82]. The frequency of myocarditis and pericarditis after vaccination with the mRNA-1273 and BNT1262b2 COVID-19 vaccines has been listed as very rare (<1 in 10,000), and these adverse events appear more frequently in younger males [81,82]. To examine whether there are differences in the underlying mechanisms of these cardiac adverse events and of the cardiac involvement in MIS-C, Amodio et al. compared the proteomic profiles of children diagnosed with MIS-C with cardiac involvement and of age-, pubertal age- and gender- matched children that were diagnosed with cardiac adverse events following COVID-19 mRNA immunization (probable or confirmed myocarditis, acute pericarditis) [45]. Healthy controls and patients with SARS-CoV-2 infection were also included in the study [45]. Plasma proteins were measured with a PEA (Inflammation panel of the Olink platform) [45]. Overall, in this study, children diagnosed with cardiac adverse events following COVID-19 mRNA immunization had higher levels of proteins related to myocardial injury compared the other groups, reflecting heart-restricted inflammation [45].The study also illustrated that MIS-C patients had a distinct inflammatory profile from children diagnosed with post-COVID-19-vaccination cardiac adverse events [45]. More specifically, in comparison with these children, MIS-C patients had higher levels of some proinflammatory cytokines that are related to systematic inflammation (e.g., CXCL9, CXCL10, IFN-γ) (Table 3) [45].

## 11. Limitations and Challenges in Proteomic Research for MIS-C Biomarkers

Despite the opportunities that proteomics provides in the search for specific and sensitive diagnostic and prognostic biomarkers for MIS-C, there are some limitations and challenges in this research field that must be considered. Firstly, a limitation of most of the reviewed studies was the relatively small number of MIS-C cases. It should be noted that MIS-C appears to be a rare syndrome, with its incidence initially estimated at 45–54 cases/100,000 SARS-CoV-2 infections in children < 15 years old [83]. Its incidence has further declined during the SARS-CoV-2 Omicron (B.1.1.529) variant wave [83]. A possible explanation for this decrease in MIS-C rates is the emergence of *novel* SARS-CoV-2 variants with a reduced ability to trigger an exaggerated inflammatory response [11,83]. It has also been suggested that COVID-19 vaccination may be associated with the reduced incidence of the syndrome [11,83,84]. The decreasing incidence of the syndrome is a significant challenge in the proteomic research for MIS-C biomarkers, also because candidate biomarkers need to be validated in prospective cohorts of patients in order to be implemented in daily clinical practice [16]. Only a small number of the reviewed studies proceeded to the validation of their proteomic findings in independent prospective cohorts using different methodologies (e.g., ELISA). Additionally, due to the small number of the reviewed studies and their heterogeneity regarding the sample types, the proteomic assays used, the number of patients, the type of control groups and the timing of sample acquisition (before or after immunomodulatory treatment) from MIS-C patients and their pediatric hyperinflammatory controls, we were able to identify similarities in the proteomic findings only across a small number of them.

## 12. Future Directions

In spite of its declining incidence since the start of the COVID-19 pandemic, MIS-C continues to occur sporadically, and it is unknown if it could potentially resurge as new SARS-CoV-2 variants emerge and vaccine-induced immunity wanes [11]. Also, given that the timely recognition of MIS-C and the prompt treatment of the affected children are essential for better patient outcomes, we believe that ongoing monitoring and the investigation of this severe COVID-19 complication are still important [83]. The research for MIS-C biomarkers also remains relevant, as it may additionally provide insights into the underlying molecular pathways of other hyperinflammatory pediatric conditions such as KD and MAS, contributing to the identification of diagnostic biomarkers and therapeutic targets for these conditions as well [83].

Proteomic technologies hold great promise in the search for MIS-C biomarkers. The continuing advances in proteomic techniques such as improved instrumentation, advancements in sample preparation methods and data analysis (including AI and machine learning approaches) and increased availability of high-throughput affinity-based proteomic assays will further improve the turnaround time, the proteomic depth and the accuracy of proteomic studies of MIS-C [12,16,85]. Furthermore, to overcome the limitations of small sample sizes due to the decreasing incidence of MIS-C, multi-centered collaborative proteomic studies could be designed in the future. Larger prospective studies focusing on the validation of the candidate biomarkers identified by the reviewed studies should be conducted. Moreover, some of the included studies in this present review applied a multi-omics approach to unravel the complex immunopathology of the syndrome [39,40,41,42]. These studies provided further insights into the pathogenesis of the syndrome. For example, Druzac et al., by also applying a metabolomic and lipidomic analysis in samples from patients with MIS-C, SARS-CoV-2 infection and healthy controls, found alterations in the absorption and metabolism of nutrients and observed elevations of inflammatory mediators. In this study, the elevation of metabolites like lactic acid and inflammatory mediators (20-HETE, 9-HpODE) appeared to be characteristic of MIS-C, while other analytes (e.g., DHA, aconitic acid) were found to be significantly decreased in MIS-C. Future proteomic research in MIS-C should also apply a multi-omics approach, as the integration of proteomic findings with genomic, epigenomic, transcriptomic, lipidomic and metabolomic data provides a more complete understanding of the underlying disease pathogenesis at a molecular level [85]. Finally, proteomic studies that incorporate patients fulfilling the revised (2023) CDC MIS-C case definition [23] are also needed, as they might be able to better recognize more distinct differences in the proteomic profile of MIS-C compared to its clinical mimics.

## 13. Conclusions

In the current review, we focused on the proteomic research of MIS-C and summarized the key findings of studies that compared the proteomic profiles of MIS-C patients, with conditions that mimic MIS-C. We focused on studies using either Mass Spectrometry or affinity-based proteomic methods (Aptamer-based or Proximity Extension proteomic assays). Collectively, according to our findings, MIS-C appears to be a complex inflammatory condition, during which several biological processes seemed to be affected, including the pathways of inflammatory responses, angiogenesis, lipid metabolism and the coagulation and complement cascades. Depending on the method and the control group, different proteins were increased or decreased in the MIS-C group, with only partial agreement among them. Although proteomic research in MIS-C appears to be promising for understanding the pathogenesis and uncovering candidate biomarkers, more proteomic studies are needed to recognize and validate biomarkers that could be applied in clinical settings to discriminate MIS-C from similar clinical syndromes.

## Figures and Tables

**Table 1:** Main clinical features of MIS-C, classic Kawasaki Disease, pediatric sepsis and septic shock, Macrophage Activation Syndrome (MAS) and Staphylococcal or Streptococcal Τoxic Shock Syndrome (TSS).

	MIS-C 2023 CDC Case Definition ^a^	Classic Kawasaki Disease 2017 AHA Criteria ^b^	MAS Characteristic Clinical Features ^c^	Pediatric SIRS, Sepsis, Septic Shock ^d^	CDC Definitions of Staphylococcal or Streptococcal TSS ^e^
**Fever**	Yes *(≥38.0 °C)*	Yes *(for at least 5 days)*	Yes *(high fever, non-remitting)*	Hyperpyrexia (*>38.5 °C*) Or hypothermia *(<36 °C*)	Yes (*Staph. TSS:* ≥*38.9 °C*)
Main Organ/System Involved *	Cardiac, Gastrointestinal, Hematologic, Mucocutaneous	Lymph nodes, Mucocutaneous *(Cardiac involvement is not included in the criteria of classic KD, however coronary artery aneurysms are reported in approximately 25% of untreated cases)*	CNS, Hematological, Hepatic, Mucocutaneous, Spleen	Cardiovascular, CNS, Hematologic, Hepatic, Kidney, Respiratory	*CNS (Staph. TSS), Hepatic, Hematologic, Gastrointestinal (Staph. TSS), Kidney, Muscular (Staph. TSS), Mucocutaneous,* *Respiratory (Strep. TSS)*
**Gastrointestinal** **Symptoms**	Yes (*abdominal pain or* *vomiting or* *diarrhea*)	-	-	-	Yes *(Staph. TSS:* Vomiting, diarrhea)
**Cardiac** **involvement**	Yes *(LVEF < 55% or* *CAA or* *Elevated Troponin)*	- *(Cardiac involvement is not included in the criteria of classic KD, however coronary artery aneurysms are reported in approximately 25% of untreated cases)*	-	Yes (*SIRS: Age-dependent tachycardia or bradycardia;* *Severe sepsis: cardiovascular dysfunction defined as hypotension, receipt of vasoactive medication or impaired perfusion despite fluid resuscitation*)	-
**Respiratory** **Involvement**	-	-	-	Yes (*tachypnea or * *need for mechanical ventilation or respiratory dysfunction*)	Yes *(Strep.TSS: ARDS)*
**Lymphadenopathy**	-	Yes *(cervical)*	Yes *(generalized)*	-	-
**Mucocutaneous** **involvement**	Yes (*rash or Inflammation of the oral mucosa or conjunctivitis or conjunctival injection or extremity findings*)	Yes (*rash or bilateral conjunctival injection or oral mucosal changes or* *extremity findings*)	Yes (*rash, mucosal bleeding*)	-	Yes *(Staph: rash, vaginal, oropharyngeal, or conjunctival hyperemia;* *Strep TSS: rash*,*soft-tissue necrosis)*
**Rash Characteristics**	Any type of rash	Polymorphous rash (*maculopapular, diffuse erythroderma, or erythema multiforme-like*)	Petechiae, Purpuric [24]	-	Staph. TSS: diffuse macular erythroderma Strep. TSS: generalized erythematous macular rash, soft-tissue necrosis
**Hematologic Involvement**	Yes (*platelet count < 150,000 cells/µL or absolute lymphocyte count < 1000 cells/µL*)	-	-	Yes (*SIRS: leukocyte count elevated or depressed for age or >10% immature neutrophils,* *hematologic dysfunction*)	Yes *(Staph. TSS:* platelet Count *< 150,000 cells/µL* *Strep. TSS: coagulopathy)*
**CNS** **Involvement**	-	-	Yes	Yes (*neurological dysfunction: altered mental status or * *Glasgow Coma Scale ≤ 11*)	Yes (*Staph. TSS:* disorientation or alterations in consciousness without focal neurologic signs)
**Hepatic** **Involvement**	-	-	Yes *(hepatomegalia)*	Yes *(hepatic dysfunction)*	Yes (*liver abnormalities*)
**Kidney** **Involvement**	-	-	-	Yes (*oligouria or renal dysfunction*)	Yes (*renal abnormalities*)
**Shock**	Yes (*presence of shock is * *included in the clinical criteria for MIS-C*)	Uncommon [*incidence rate of Kawasaki Disease Shock Syndrome: 2.60–6.95%* [25]*;* *Shock is not in the diagnostic criteria for classic Kawasaki Disease*]	Yes *[many patients present with shock and require ICU admission* [26]]	Yes (*pediatric septic shock*)	Yes (*hypotension/shock)*

Notes: The main clinical features presented in Table 1 are derived from the definitions for the diagnosis of MIS-C, classic Kawasaki Disease, pediatric sepsis septic shock and Staphylococcal or Streptococcal TSS. The proposed laboratory criteria for the diagnosis of MAS in patients with systemic Juvenile Idiopathic Arthritis according to the “2016 Classification Criteria for Macrophage Activation Syndrome Complicating Systemic Juvenile Idiopathic Arthritis” are the following: Ferritin > 648 ng/mL, and any of the two following: Platelet count ≤ 180.000 cells/µL, AST > 48 IU/L, Triglycerides > 1.76 mmol/L, Fibrinogen ≤ 10.6 μmol/L [27].,and the most characteristic clinical features of MAS are outlined in Table 1 [27]. * Refers to the organ/systems that are usually involved in each clinical entity. Not all organ/systems are required to be involved concurrently for the establishment of the diagnosis according to the case definitions of MIS-C, classic KD, septic shock, TSS. ^a^ The clinical criteria for MIS-C from the updated 2023 CDC MIS-C case definition were used [23]. ^b^ The 2017 American Heart Association (AHA) diagnostic criteria for classic Kawasaki Disease are presented [28]. ^c^ The diagnosis of MAS in patients with systemic Juvenile Idiopathic Arthritis according to the “2016 Classification Criteria for Macrophage Activation Syndrome Complicating Systemic Juvenile Idiopathic Arthritis” is based on the following laboratory criteria: Ferritin > 648 ng/mL, and any of the two following: Platelet count ≤ 180.000 cells/µL, AST >48 IU/L, Triglycerides > 1.76 mmol/L, Fibrinogen ≤ 10.6 μmol/L [27]. ^d^ Definitions according to the 2005 International Pediatric Sepsis Definition Consensus conference [29,30]. ^e^ According to the Toxic Shock Syndrome (other than Streptococcal) (TSS) 2011 CDC Case Definition [31] and the 2010 Definition of Streptococcal Toxic Shock Syndrome (STSS) 2010 CDC case Definition [32]. Abbreviations: MIS-C: Multisystem Inflammatory Syndrome in Children Associated with COVID-19, CNS: Central Nervous System, SIRS: Systematic Inflammatory Response Syndrome, LVEF: Left Ventricular Ejection Fraction, CAA: Coronary Artery Abnormalities, ARDS: Acute Respiratory Distress Syndrome, ICU: Intensive Care Unit.

**Table 2:** Characteristics of proteomic studies in MIS-C patients using Mass Spectrometry (MS) or affinity-based methods.

First Author (Year)	Study Population	Sample Size	Enrollment Period of MIS-C Patients (*Month/Year*)	Specimen Type	Proteomic Method	No of MIS-C Patients ^c^	Timing of MIS-C Specimen Acquisition: (*Before or After MIS-C Treatment*)
Consiglio (2020) [37]	MIS-C, HC ^a^, KD acute SARS-CoV-2 infection ^a^	101	March–May/2020	Plasma	Olink Immune Response and Inflammation Panels	11	Before Or After ^d^
Gruber (2020) [43]	MIS-C, HC ^a^ acute and convalescent SARS-CoV-2 infection (children and adults)	24 ^b^	April–June/2020	Plasma	Olink Inflammation panel	9	Before Or After ^d^
Diorio (2021) [38]	MIS-C, acute SARS-CoV-2 infection ^a^, HC ^a^	88	April–October/2020	Plasma	Olink Explore 1536/384 panel	22	Before Or After ^d^
Porritt (2021) [39]	MIS-C, KD, HC, Febrile controls ^a^	96	NA	Plasma or Serum	LC-MS/MS	25	Before Or After
Ramaswamy (2021) [40]	MIS-C, HC ^a^, HC (adults), acute SARS-CoV-2 infection (adults)	7 ^b^	NA	Serum	SomaScan platform (v4)	3	After
Yonker (2021) [44]	MIS-C, HC ^a^, acute SARS-CoV-2 ^a^	100	NA	Plasma	Multiplexed Quantitative LC-MS Proteomics	13	Before
McCafferty (2022) [9]	MIS-C, HC ^a^, COVID-19- ARDs ^a^,	54	2020	Plasma	RP-HPLC-MS	29	Before Or After (*in most patients*)
Amodio (2023) [45]	MIS-C, c-AEFI, HC (*age-matched*) acute SARS-CoV-2 infection (*age-matched*)	81	NA	Plasma	Olink Inflammation panel	14	Before
Sacco (2022) [41]	MIS-C, acute SARS-CoV-2 infection ^a^, HC ^a^	262	03/2020–02/2021	Plasma	SomaScan platform	19	Before Or After (*in most patients*)
Brodeur (2023) [46]	MIS-C, KD, nsJIA, sJIA, JDM, MAS, HC ^a^, Febrile controls ^a^	215	01/2020–12/2022	Plasma or Serum	Olink Target 48 Cytokine Panel	25	Before
Druzak (2023) [42]	MIS-C, HC ^a^, HC (adults), Critically-ill adults without SARS-CoV-2 infection, acute SARS-CoV-2 infection (children and adults)	19 ^b^ (*children*)	NA	Plasma	LC-MS/MS	5	Before Or After
Nygaard (2024) [11]	MIS-C, KD, viral or bacterial infection, sepsis	94	04/2020–03/2022	Plasma	LC-MS/MS	27	Before (*in most patients*) Or After ^d^
Patel (2024] [10]	MIS-C, HC ^a^, SARS-CoV-2 negative sepsis	36	NA	Plasma	Olink Explore 3072 library	12	NA
Reiter (2024) [5]	MIS-C, KD, HC ^a^, pediatric hyperinflammation (sJIA-assosiated MAS, secondary HLH)	65	NA	Serum	Olink Target 96 Inflammation and Target 96 Cardiovascular III panel	31	Before ^d^
Tulling (2024) [47]	MIS-C, HC^a^ (*age-matched*)	248	03/2020–04/2023	Serum	Custom 60-plex Luminex panel Olink Explore 3072 panel	60 ^e^	Before Or After

Notes: ^a^ Refers to children, ^b^ Subset of study participants whose samples were used for proteomic analysis, ^c^ Refers to the number of MIS-C patients whose serum or plasma samples were obtained for proteomic analysis, ^d^ For some patients, both pre- and post-treatment samples were available for proteomic analysis, ^e^ In the study by Tulling et al. [47], 43 MIS-C patient samples were analyzed with the Custom 60-plex Luminex panel, and 17 were analyzed using the Olink Explore 3072 panel. Abbreviations: MIS-C; Multisystem Inflammatory Syndrome in Children Associated with COVID-19, KD; Kawasaki Disease, HC; Healthy Control, NA; not available, LC-MS/MS; Liquid Chromatography—Tandem Mass Spectrometry, ARDs; Acute Respiratory Distress Syndrome, RP-HPLC-MS: Reverse phase high-performance LC-MS, c-AEFI; cardiac adverse events following COVID-19 immunization, ns-JIA; nonsystematic Juvenile Idiopathic Arthritis, sJIA; systematic JIA, JDM; Juvenile Dermatomyositis, MAS; Macrophage Activation Syndrome, HLH; Hemophagocytic Lymphohistiocytosis.

**Table 3:** Key findings of proteomic studies using Mass Spectrometry (MS) or affinity-based methods to identify differences in the serum or plasma proteomes of MIS-C patients and patients with other pediatric inflammatory conditions.

MIS-C vs. Control Group	Reference	Proteomic Method	Proteins Increased (↑) in MIS-C Compared to Control Group	Proteins Decreased (↓) in MIS-C Compared to Control Group	Biological Pathways
**1.MIS-C vs.** **Acute** **SARS-CoV-2** **infection**	Gruber et al. [43]	Affinity-based	↑ CXCL5, CXCL11, CXCL1, CXCL6, IL-17A, CD40, IL-6 *	NA	NA
	Diorio et al. [38]	Affinity-based	↑ NT-PROBNP, PLA2GA, CALCA, CXCL10, CXCL9, CCL7, IL1RL1, NPPB, REG3A, TNFRSF6B, VSIG4, IL2RA, IL5RA, PLAT, REG1B, SDC1, SIGLEC10 (*CG: mild SARS-CoV-2 infection*) ↑ CXCL9, MMP8, OSM, RNASE3, AZU1, CALCA, CXCL10, IL10, MMP9, NTproBNP (*CG: severe SARS-CoV-2 infection*)	↓ SFRP1, FGF21, PSPN, PBLD, GSTA1, FABP1, DDX58, AGR2, BPIFB1, GAL, TRIM21 (*CG: severe SARS-CoV-2 infection*)	NA
	Yonker et al. [44]	MS	↑ zonulin, LBP	NA	NA
	Sacco et al. [41]	Affinity-based	↑ NPPB.1, PLA2G2A, H2AFZ, HIST3H2A, IGFBP2, IL22, FERRITIN, CD177, PRTN3, APOE HGE, HIST2H2BE, MRC1, IL1R2, PLAT, RETN, MMP9, FCGR3B, CCL23, TNFRSF1B, SELE, FSTL3, CD163, MMP17, TNFRSF1A (*Top 25 upregulated*)	↓ CA6, APOM, PGAM1, MMP12, IL22RA2, SLITRK5, IGFBP1, IGFBP3, CADM1, CD36, IL1R1, CTSV, WIF1, SPINT1, MRC2, ADAMTS13, FAP, ACAN, EPHA5, AHSG, FETUB, GDF2, CNTFR, RET, HPX (*Top 25 downregulated*)	Hyperactivation of matrisome-associated response, cell activation, signaling receptor binding, defense response, collagen-containing extracellular matrix, locomotion, external encapsulating structure, positive regulation of signaling, cell migration, biological adhesion, locomotion, cell surface, signaling receptor binding neurogenesis, peptidyl tyrosine modification neuron development, cell part morphogenesis, neuron differentiation
	Druzak et al. [42]	MS	↑ CRP, SAA1, DEFA 1: DEFA:1B, THBS1, PPBP, WARS1, GSTO1, LBP, VWF, ELANE	↓ IGFBP, APOM, AHSG, APOA1, ITIH2, ITIH1, HRG	pCOVID-19; various infectious processes (e.g., coronavirus infection), metabolic processes associated with nucleotides and proteins. MIS-C; African trypanosomiasis, Metabolic processes associated with lipid metabolism
	Amodio et al. [45]	Affinity-based	↑ IL-10RB, CXCL9, IL-17A, VEGFA, FGF-23. SLAMF1, CSF-1, GDNF	↓ LAP TGF-beta-1, SCF	NA
2. MIS-C vs. **COVID-19 ARDS**	McCafferty et al. [9]	MS	*Samples of MIS-C and ARDS did not separate distinctly*		NA
**4.MIS-C vs.** **Kawasaki Disease**	Consiglio et al. [37]	Affinity-based	↑ ADA, SCF, TWEAK (*among others*)	↓ IL-6, IL-17A, CXCL10, DCBLD2 (*among others*)	NA
	Porritt et al. [39]	MS	↑ FTL, FCGR3A, C1qA, C1qB, C1qC, TNC, QSOX1, GPX3, PRSS3, AZGP1, SERPINB3, RPS11 (*in severe MIS-C*)	↓ HRG, SHBG, C7, A2M (*in severe MIS-C*)	NA
	Brodeur et al. [46]	Affinity-based	↑ IL-10, CCL4, CCL8, CXCL9, IFN-γ	↓ IL-17 cytokine family (IL-17A, IL-17C, and IL-17F), IL-13	NA
	Reiter et al. [5]	Affinity-based	↑4E-BP1, Gal-3, TIMP4, ADA, TR-AP, SIRT-2 (top 6 markers)	NA	NA
**5.MIS-C vs.** **HLH/MAS**	Brodeur et al. [46]	Affinity-based	↑ IL-10, CCL4, CCL8	↓ IL-33, IL-18	NA
	Reiter et al. [5]	Affinity-based	58 protein markers could be identified, the following of which had ↑ levels, among others: IL-17A, MMP-9, HGF, CASP-3	58 protein markers could be identified, the following of which had ↓ levels, among others: IL-18, TRANCE	NA
6. MIS-C vs. **Bacterial or Viral Infection, Sepsis and Kawasaki Disease**	Nygaard et al. [11]	MS	Significantly different levels of 105 proteins were identified, the following of which had ↑ levels: LCP1, FCGR3A (CD16a), B2M, FCGBP, SERPINA3, CRP, ORM1/2, SAA1, HP, CD14, C1QB, C1S, C2, C4BPB, C4B, C9, CFI, FCN2, F10, F11, FG, vWF, APOE, APOF (*among others*)	Significantly different levels of 105 proteins were identified, the following of which had ↓ levels: PGLYRP2, F12, F13A1, F13B, SERPINC1, PROC, SERPIND1, PF4, KLKB1, PLG, THBS1, PPBP, ACTB, ECM1, FN1, CLU, APOA, APOC1, APOC3, APOH, BCHE (*among others*)	15 pathways affected in MIS-C compared to controls including pathways of immunological responses, coagulation, cell death and cell growth, platelet activation
7. MIS-C vs. **Bacterial or Viral Infection**	Nygaard et al. [11]	MS	↑ LCP1, CD16a, SERPINA3 (*part of a 4-protein diagnostic signature developed with artificial intelligence*)	↓ BCHE (*part of a 4-protein diagnostic signature developed with artificial intelligence*)	NA
8. MIS-C vs. **Pediatric Sepsis**	Patel et al. [10]	Affinity-based	↑ LTA4H, C3, PDGFA, F10, ANGPT1, PPBP, BDNF, SERPINI1, EGF, LYSMD3 (*part of a 15-protein set identified with COMBAT-Seq batch effect adjusted feature selection*)	↓ MRPL58, BTLA, CREBZF, PTN, BMP4 (*part of a 15-protein set identified with COMBAT-Seq batch effect adjusted feature selection*)	Inflammation, cell growth and survival metabolism, angiogenesis, organ/cell-specific function
9. MIS-C vs. **c-AEFI**	Amodio et al. [45]	Affinity-based	↑ IL-10, CXCL10, CXCL9, CDCP1, IFN-γ, MCP3, TGF-α	↓ CXCL5, SCF, CD244, IL-20RA, FGF-5	NA

Notes: * The study by Gruber et al. compared the proteomic profiles of children with MIS-C with the proteomic profiles of both children and young adults with acute SARS-CoV-2 infection. The findings of the study presented in Table 1 refer to the comparison of the proteomic profile of children with MIS-C and children with acute SARS-CoV-2 infection. Abbreviations: MIS-C; Multisystem Inflammatory Syndrome in Children Associated with COVID-19; NA; Not available; pCOVID-19; pediatric COVID-19; ARDS; Acute Respiratory Distress Syndrome; MAS; Macrophage Activation Syndrome; HLH; Hemophagocytic Lymphohistiocytosis c-AEFI; cardiac adverse events following COVID-19 immunization.

## Data Availability

No new data were created or analyzed in this study. Data sharing is not applicable to this article.

## Supplementary Materials

The following supporting information can be downloaded at https://www.mdpi.com/article/10.3390/children11101174/s1; Table S1: Key Findings of proteomic studies using Mass Spectrometry (MS) or affinity-based methods to identify differences in the serum or plasma proteomes of MIS-C patients and healthy controls.
