# Supplementary material for: Proteomic Signatures of Multisystem Inflammatory Syndrome in Children (MIS-C) Associated with COVID-19: A Narrative Review
Source: Children (Basel). 2024 Sep 26;11(10):1174. doi: 10.3390/children11101174 (PMC11505985; doi:10.3390/children11101174)
Supplement: Supplementary file 1 [file children-11-01174-s001.zip › children-3199045-supplementary.pdf]

Supplementary Table 1. Key Findings of proteomic studies using Mass Spectrometry (MS)- or Affinity-Based methods to identify differences in the serum or plasma proteomes of MIS-C patients and Healthy Controls.

Reference	Proteomic Assay	Proteins increased (↑) in MIS-C compared to Healthy Controls	Proteins decreased (↓) in MIS-C compared to Healthy Controls	Biological Pathways
Gruber et al. [1]	Affinity-based	↑OPG, IL-16, IL-18, IL-10RA, CDCP1, CCL19, IL-15A, CXCL10, CCL4, CCL3, EN-RAGE, CSF-1,LIFR,IL-18R1,PDLI, HGF,IL-17A, CCL28,CCL20	NA	Pro-Inflammation, T-cell and NK-cell modulation and chemotaxis, Monocyte and Neutrophil function, Immunosuppression, Mucosal immunity
Diorio et al. [2]	Affinity-based	↑ PLA2G2A, MPO, IL1RL1, VSIG4, CXCL10, CALCA, GNLY, FKB5, WARS, PRM2, AZU1, MMP8, CCL7, NTproBNP, CLEC5A, LILRB4, TYMP, CXCL9, CXCL11, OSM, TIMP3, IL6, APEX1, PFKB2, GZMB, MNDA, RNASE3, REG3A	↓ BPIFB1, CCN1, CLEC4C, CRH, DRAXIN, FRZB, GAL, GDF2, NPPC, OGN, SMAD5, ITGA11, KITLG, SERPINA9, TNFSF11, CRH	Apoptosis, Viral Protein Interaction with Cytokine-to-cytokine receptor, Pathogenic *Escerichia coli* Infection, TNF Signaling Pathway
Porritt et al. [3]	MS	244 proteins were significantly ↑ in severe MIS-C: SAA1, CRP, CARN1, LBP, CDC5L, S100A9, AQR, PSIP1, PYGB, LRG1, HP, ORM1, CCDC58, HIST1H4A, SERPINA3, S100AB, RAB21, RPS7, HLA-DRA, GAPDH, SND1, HBB, ACP1, HBA1, FGB *(Top upregulated)*	135 proteins were significantly ↓ in severe MIS-C: SHBG, AKR1A1, IGFBP3, GPLD1, SERPINA5, LAMC1, RPL15, IGFALS, CCT7, SERPINA4, PCMT1, RBP4, SHMT1, VDAC1,TTR, GATM, GLOD4, DDX6, APOL2, PGLYRP2, BGN,TALDO1, TPP1, APOA4, APOA1 *(Top downregulated)*	Humoral immune response, Complement pathways, Platelet activation and Coagulation pathways, Fc receptor signaling, neutrophil-mediated responses, Phagocytosis pathways, VEGF signaling, Smooth muscle cell contraction, Lipid transport, Lipid metabolic processes, Lipoprotein clearance, regulation of body fluids, Relaxation of cardiac muscle
Ramaswamy et al. [4]	Affinity-based	↑ IL-1RN, CRP, PRSS2, FTH1 FTL, SAA1, HAMP, REG3A, TNFRSF1B, SAA2, FCGR3B, PLA2G2A, CD163, LILRA5 *(among other proteins)*	↓IL-R1 *(among other proteins)*	Enrichment of Cytokine-cytokine receptor interaction, Fluid shear stress and atherosclerosis, Complement and coagulation cascades, Prostate cancer, Axon guidance, TNF signaling pathway, Necroptosis, Hematopoietic cell lineage, Th17 cell differentiation, P13K-Akt signaling pathway
Yonker et al. [5]	MS	↑ zonulin, LBP	NA	NA
McCafferty et al. [6]	MS	85 proteins were differentially expressed between the MIS-C and Healthy groups: 52 proteins were ↑	85 proteins were differentially expressed between the MIS-C and Healthy groups: 33 proteins were ↓	Reactome: Creation of C4 and C2 activators, Classical antibody-mediated complement activation and Fc γ Receptor (FCGR) activation; STRING: Cholesterol metabolism, Complement and coagulation cascades, Prion disease
Amodio et al. [7]	Affinity-based	↑ CXCL10, CXCL9, IL-6, CXCL11, CASP-8, SIRT2, IFN-γ, IL-10, CDCP1, CCL19, MCP-3, TNF	↓ CXCL5, MMP-1, IL-7, CXCL1, CD244, TRANCE, SCF, CCL25	NA
Sacco et al. [8]	Affinity-based	↑ 242 proteins including: SAA1, CRP, PLA2G2A, NPPB.1, FERRITIN, CXCL10, sST2/sIL-33R, PRSS2, H2AFZ, IGFBP2, CHI3L1, CD177, IBSP, HIST3H2A, SIGLEC14, PRTN3, IL18BP, HAMP, CCL23, MRC1, IL1R2, CXCL9, HIST2H2BE, TNNT2, TNFRSF1B *(Top 25 upregulated)*	↓ 158 proteins including PPIF, EIF4G2, PRKACA, ENO2, PRKCA, KPNB1, CA6, CFP, GRB2, TPM4, CASP3, PDPK1, DUSP3, LYN, ADRBK1, XPNPEP1, FYN, LYN.1, SNX4, PTPN11, NME2, RAC1, AKT2, DAPK2, TPT1 *(Top 25 downregulated)*	Matrisome Activation, Defence Response, Receptor Binding Signaling, Cell Activation, Cytokine-Cytokine Receptor Interaction, Cytokine-mediated signaling pathway, Regulation of Immune System Process, Cell Population Proliferation, Protein Phosphorylation, Reactome signaling by receptortyrosine kinases, Biological adhesion, Regulation of

				phosphorus metabolic process, Regulation of response to external stimulus, Response to cytokine, Peptidyl amino acid modification, Reactome cytokine signaling in immune system, Regulation of response to stress
Druzak et al. [9]	MS	↑ CRP, SAA1, DEFA 1: DEFA:1B, THBS1, PPBP, WARS1, GSTO1, LBP,VWF, ELANE	↓ IGFBP3, APOM, AHSG, APOA1, ITIH2, ITIH1, HRG	KEGG Pathways: African Trypanosomiasis Transcriptional Phagosome, Thyroid Hormone Synthesis, misregulation in cancer, Lipid and atherosclerosis, Fat digestion and absorption, Fluid shear stress, Tuberculosis ECM Receptor Interaction, omplement and coagulation cascades, inflammatory processes (such as IL-17A signaling, various infections), autoimmune disease
Patel et al. [10]	Affinity-based	*58 protein-model separated MIS-C from Healthy Controls*		Inflammation, Cell growth and survival, Metabolism, Angiogenesis, Organ/cell-specific functions.

Abbreviations: MIS-C; Multisystem Inflammatory Syndrome in Children Associated with COVID-19, NA; Not available MS; Mass Spectrometry.

1. Gruber, C.N.; Patel, R.S.; Trachtman, R.; Lepow, L.; Amanat, F.; Krammer, F.; Wilson, K.M.; Onel, K.; Geanon, D.; Tuballes, K.; et al. Mapping Systemic Inflammation and Antibody Responses in Multisystem Inflammatory Syndrome in Children (MIS-C). *Cell* **2020**, *183*, 982-995.e914, doi:10.1016/j.cell.2020.09.034.
2. Diorio, C.; Shraim, R.; Vella, L.A.; Giles, J.R.; Baxter, A.E.; Oldridge, D.A.; Canna, S.W.; Henrickson, S.E.; McNerney, K.O.; Balamuth, F.; et al. Proteomic profiling of MIS-C patients indicates heterogeneity relating to interferon gamma dysregulation and vascular endothelial dysfunction. *Nat Commun* **2021**, *12*, 7222, doi:10.1038/s41467-021-27544-6.
3. Porritt, R.A.; Binek, A.; Paschold, L.; Rivas, M.N.; McArdle, A.; Yonker, L.M.; Alter, G.; Chandnani, H.K.; Lopez, M.; Fasano, A.; et al. The autoimmune signature of hyperinflammatory multisystem inflammatory syndrome in children. *J Clin Invest* **2021**, *131*, doi:10.1172/jci151520.
4. Ramaswamy, A.; Brodsky, N.N.; Sumida, T.S.; Comi, M.; Asashima, H.; Hoehn, K.B.; Li, N.; Liu, Y.; Shah, A.; Ravindra, N.G.; et al. Immune dysregulation and autoreactivity correlate with disease severity in SARS-CoV-2-associated multisystem inflammatory syndrome in children. *Immunity* **2021**, *54*, 1083-1095.e1087, doi:10.1016/j.immuni.2021.04.003.
5. Yonker, L.M.; Gilboa, T.; Ogata, A.F.; Senussi, Y.; Lazarovits, R.; Boribong, B.P.; Bartsch, Y.C.; Loiselle, M.; Rivas, M.N.; Porritt, R.A.; et al. Multisystem inflammatory syndrome in children is driven by zonulin-dependent loss of gut mucosal barrier. *Journal of Clinical Investigation* **2021**, *131*, doi:10.1172/jci149633.
6. McCafferty, C.; Cai, T.; Borgel, D.; Lasne, D.; Renolleau, S.; Vedrenne-Cloquet, M.; Bonnet, D.; Wu, J.; Zaw, T.; Bhatnagar, A.; et al. Pathophysiological pathway differences in children who present with COVID-19 ARDS compared to COVID -19 induced MIS-C. *Nat Commun* **2022**, *13*, 2391, doi:10.1038/s41467-022-29951-9.
7. Amodio, D.; Pascucci, G.R.; Cotugno, N.; Rossetti, C.; Manno, E.C.; Pighi, C.; Morrocchi, E.; D'Alessandro, A.; Perrone, M.A.; Valentini, A.; et al. Similarities and differences between myocarditis following COVID-19 mRNA vaccine and multiple inflammatory syndrome with cardiac involvement in children. *Clin Immunol* **2023**, *255*, 109751, doi:10.1016/j.clim.2023.109751.
8. Sacco, K.; Castagnoli, R.; Vakkilainen, S.; Liu, C.; Delmonte, O.M.; Oguz, C.; Kaplan, I.M.; Alehashemi, S.; Burbelo, P.D.; Bhuyan, F.; et al. Immunopathological signatures in multisystem inflammatory syndrome in children and pediatric COVID-19. *Nat Med* **2022**, *28*, 1050-1062, doi:10.1038/s41591-022-01724-3.
9. Druzak, S.; Iffrig, E.; Roberts, B.R.; Zhang, T.; Fibben, K.S.; Sakurai, Y.; Verkerke, H.P.; Rostad, C.A.; Chahroudi, A.; Schneider, F.; et al. Multiplatform analyses reveal distinct drivers of systemic pathogenesis in adult versus pediatric severe acute COVID-19. *Nature Communications* **2023**, *14*, doi:10.1038/s41467-023-37269-3.
10. Patel, M.A.; Fraser, D.D.; Daley, M.; Cepinskas, G.; Veraldi, N.; Grazioli, S. The plasma proteome differentiates the multisystem inflammatory syndrome in children (MIS-C) from children with SARS-CoV-2 negative sepsis. *Molecular Medicine* **2024**, *30*, doi:10.1186/s10020-024-00806-x.
